# Retraction: Predictability of combined cataract surgery and trabeculectomy using Barrett Universal Ⅱ formula

**DOI:** 10.1371/journal.pone.0305361

**Published:** 2024-06-06

**Authors:** Kei Iijima, Kazutaka Kamiya, Yoshihiko Iida, Masayuki Kasahara, Nobuyuki Shoji, the PLOS ONE Editors

Following the publication of this article [1], the first author contacted *PLOS ONE* to inform the journal that this study was in breach of their institutional ethics policies. Specifically, the first author stated that data collection for this study started before the institutional ethics committee had approved the study.

The *PLOS ONE* Human Subject Research policies in place at the time this article was submitted state that researchers submitting studies involving human participants must obtain prior approval for human subjects research by an institutional review board (IRB) or equivalent ethics committee(s) and declare compliance with ethical practices upon submission. The authors’ statement that data collection started before institutional ethics approval was granted is a breach of *PLOS ONE’s* Human Subject Research policies.

In light of the concerns that this study was not conducted in line with institutional ethics policies and *PLOS ONE*’s Human Subject Research policies, the authors and the *PLOS ONE* Editors retract this article.

All authors agree with the retraction and apologize for the issues with the published article.
